# NK Cells Contribute to Protective Memory T Cell Mediated Immunity to *Chlamydia muridarum* Infection

**DOI:** 10.3389/fcimb.2020.00296

**Published:** 2020-06-17

**Authors:** Hong Wang, Jing Li, Xiaojing Dong, Xaoqing Zhou, Lei Zhao, Xiao Wang, Rasheduzzaman Rashu, Weiming Zhao, Xi Yang

**Affiliations:** ^1^Department of Pathogenic Biology & Key Laboratory of Infection and Immunity of Shandong Province, School of Basic Medical Sciences, Shandong University, Jinan, China; ^2^Institute of Basic Medical Science, Qilu Hospital of Shandong University, Jinan, China; ^3^Department of Pathology, School of Basic Medical Sciences, Shandong University, Jinan, China; ^4^Department of Immunology and Department of Medical Microbiology, Max Rady College of Medicine, University of Manitoba, Winnipeg, MB, Canada

**Keywords:** natural killer cells, *Chlamydia*, memory T cells, secondary infection, regulatory T cells

## Abstract

We previously reported that NK cells can promote type 1 T cell immune response that is essential for protection to a primary infection of *Chlamydia muridarum*. In this study, we have investigated the contribution of NK cells to memory T cells associated immunity during chlamydial infection. We have found that NK cell depletion led to impaired production of IFN-γ by memory T cells upon re-stimulation with chlamydial antigens *in vitro*. Mice with depleted NK cells also exhibited reduced type 1 T cell recall responses, with increased production of IL-4 from CD4^+^ T cells and a lower level of *Chlamydia*-specific IgG2a titers compared to control mice. In addition, Tregs response was significantly increased in mice with NK cell depletion. Moreover, NK cell-depleted mice showed an increased bacterial loads and more severe inflammatory pathological changes than control mice. These findings indicate that NK cells contribute to protective memory T cell associated immunity to chlamydial re-infection through modulating the cytokine pattern of T cell and inhibition of Tregs response.

## Introduction

*Chlamydia* is an obligate intracytoplasmic pathogen with a tropism for epithelial cells and macrophages. Several diseases caused by chlamydial infection in humans are a serious worldwide public health burden (Brunham and Rey-Ladino, 2005). Currently, there is no available vaccine for preventing chlamydial diseases. A better understanding of the mechanism of immune response in chlamydial primary and secondary infection is essential for the rational design of a successful vaccine. Findings from humans and experimental animal model have demonstrated that CD4^+^T cells, especially Th1 type of response are the crucial component in mediating protective immunity in both primary and secondary infections with *Chlamydia* (Su and Caldwell, 1995; Williams et al., 1997; Morrison and Caldwell, 2002; Gondek et al., 2009). In particular, memory CD4^+^T cells can persist for a long time, proliferate rapidly and secrete cytokines such as IFN-γ during secondary chlamydial infection (Igietseme et al., 1993; Morrison and Morrison, 2000; Stary et al., 2015). Other immune components including antibodies and CD8^+^T cells also involved in partial protection of the host against chlamydial reinfection (Starnbach et al., 2003; Morrison and Morrison, 2005; Li and McSorley, 2015).

NK cells are a predominant component of innate immune system, and also play an important role in host to combat against chlamydial infections. As a frontline responder, NK cells can contribute to host defense by cytotoxicity and cytokine-mediated effector functions without prior sensitization (Vivier et al., 2008). Besides acting as important innate effector, NK cells can regulate adaptive immune responses during primary bacterial and viral infection settings (Lodoen and Lanier, 2006; Cook et al., 2014; Crouse et al., 2015). In primary chlamydial infection, NK cells have been demonstrated to exert immunoregulatory function in adaptive immunity. In particular, NK cells promote Th1 responses by modulating dendritic (DC) function (Jiao et al., 2011; Shekhar et al., 2015). Furthermore, we have recently reported that the protective effect of NK cells is closely related to its ability to maintain a Th1/Treg and Th17/Treg balance (Li et al., 2016). However, the role of these cells in the memory associated immunity to secondary chlamydial infection is poorly understood. Recently, several reports highlighted that NK cells contribute to the protective memory responses upon secondary infection (Alexandre et al., 2016; Habib et al., 2016; Zheng et al., 2016). For example, NK cell-depleted mice showed less resistant to *Ehrlichiae* rechallenge along with impairment of protective recall responses (Habib et al., 2016). Moreover, during *Listeria monocytogenes* re-infection, activated NK cells were the major producers of early IFN-γ and promoted protective memory CD8^+^T cell response (Alexandre et al., 2016). Furthermore, NK cell–derived IFN-γ played a necessary role in the proliferation and activation of CD8^+^T cells, especially in inducing secondary CD8^+^T cell responses against HBV (Zheng et al., 2016).

Here, we have addressed the effect of NK cells on modulating memory T cells response to respiratory infection with *Chlamydia muridarum*. We have found that NK cells rapidly expand and become activated following intranasal re-infection. NK cells depletion led to the impaired functional capability of memory CD4^+^ and CD8^+^T cells especially in IFN-γ production during specific antigen re-stimulation *in vitro* and *in vivo* during secondary infection. Enhanced Tregs and Th2 response with decreased levels of *Chlamydia*-specific IgG2a antibody titer was also found in mice with NK cell depletion. Moreover, NK cell-depleted mice showed an increased bacterial load and inflammation triggered pathological changes compared to control mice at an early stage of secondary infection. These findings indicate that NK cells contribute to protective recall response during intranasal chlamydial infection through modulating T cell immunity.

## Materials and Methods

### Organism

*Chlamydia muridarum* Nigg strain was used for this study. The culture of the organism was performed as described previously (Wang et al., 2012). The purified *C. muridarum* elementary bodies (EBs) were prepared by density gradient centrifugation and then stored at −80°C for future use. The same stock was used for all of the experiments.

### Mice

Six to eight-week-old male C57BL/6 mice were purchased from Vital River Laboratory (Beijing, China). Animal experimental studies were conducted in accordance with a protocol approved by the Animal Care and Use Committee of Shandong University.

### NK Cell Depletion

NK cells were depleted by intravenous injection with anti-asialo GM1 (Wako Chemicals, Richmond, VA). At 1 day before and 1 day after secondary infection, 20 μl anti-asialo GM1 or normal rabbit IgG (isotype control) antibodies were used and followed by 10 μl of a dose of every 3 days. The depletion efficiency of NK cells was confirmed by flow cytometric assay.

### Mice Infection Protocol and *C. muridarum* Quantification

Mice were inoculated intranasally with *C. muridarum* (1 × 10^3^ inclusion-forming units, IFUs) in 40 μl PBS, and then the secondary infection was performed with the same dose of the organism after 8 weeks of primary infection. For the determination of *C. muridarum* growth *in vivo*, mice were euthanized on predetermined days and the lungs tissues were isolated aseptically followed by homogenization in SPG buffer. After centrifugation, the supernatants of homogenates were collected for determination of the *C. muridarum* loads by infection on Hep-2 cells.

### Cell Isolation and Cytokine Detection

Mononuclear cells of spleen and lung tissues were separated as described previously (Peng et al., 2014). Briefly, spleens were aseptically removed, homogenized and treated with RBC lysis buffer (ebioscience). For the preparation of lung mononuclear cells, lung tissues were teased apart and digested with collagenase XI (1 mg/ml). After digestion, cells were resuspended in 35% Percoll solution (Pharmacia) and followed by erythrocytes lysis. All the cells were suspended with complete RPMI medium containing 10% FBS, 100 μg/ml streptomycin, 100 U/ml penicillin, and 5 × 10^−5^ M 2-mercaptoethanol. Mononuclear cells of spleen and lung (7.5 × 10^6^ and 5 × 10^6^ cells/ml, respectively) were incubated with 1 × 10^5^ IFUs/ml heat-killed (HK)-*C. muridarum* for 72 h. Cytokines secretion in the culture supernatants were tested by ELISA kit (eBioscience).

### *In vitro* Recall Response

Eight weeks after primary intranasal inoculation with *C. muridarum*, the infected animals received twice intravenous injection of anti–asialo GM1 or isotype control antibody (20 μl) with the interval of 1 day. Two more days later, the mice were killed and spleen cells were isolated. In some experiments, 2 × 10^6^/well cells were cultured with stimulation with HK-EBs (1 × 10^5^ IFUs/ml) for 72 h. At the end of the culture, cell stimulation cocktail and Brefeldin A were added, and then the expression of intracellular IFN-γ in T cells was detected using flow cytometry. IFN-γ secretion in supernatants was measured using ELISA kit. In proliferation experiments, splenocytes (1 × 10^6^/ml) were labeled with CFSE dye at a final concentration of 0.5 μM (Invitrogen), plated in 96-well plates (200 μl/well) and stimulated with HK-EBs. After 5 days, the frequency of IFN-γ expression within the proliferating cells was determined by flow cytometric assay.

### Flow Cytometry

For the analysis of expression of cell membrane proteins, cells were stained with anti-mouse antibodies including anti-CD3ε, anti-NK1.1, anti-CD69, anti-CD4, anti-CD8, anti-CD44, and anti-CD25 mAbs. For intracellular staining of Tregs, cells were further stained with anti-Foxp3 mAb after fixation/permeabilization buffer treatment. The frequencies of CD4 T cells, Tregs or their ratios at different infecting dose were shown in Figure S1. For intracellular staining of IFN-γ and IL-4, 2 × 10^6^ cells were incubated in complete RPMI-1640 medium containing cell stimulation cocktail (2 μl/well) and Brefeldin A (1 μl/well). After being incubated with cell surface antibodies, fixed and permeabilized, cells were incubated with anti-IL-4 or anti-IFN-γ mAb. All antibodies and reagents were purchased from eBioscience. The stained cells were tested by CytExpert flow cytometer and analyzed using CytExpert or flowJo software.

### Serum Antibody Analysis

Serum IgG1 and IgG2a antibodies against *C. muridarum* were measured using a routine method. After coating with HK-EBs overnight and blocking with BSA for 90 min, microtiter plates were incubated with serum samples diluted serially for 2 h. Biotinylated goat anti-mouse (Southern biotech) and AP-conjugated streptavidin (Sigma-Aldrich) were applied successively. Following the addition of *P*-nitrophenyl phosphate, the absorbance was evaluated at 405 nm by using a microtiter reader.

### Histology

The lung tissues were collected from different groups of mice and treated with paraformaldehyde for fixation and then paraffin embedding was performed. Four-micron sections were stained with H&E followed by examination according to the criteria as described elsewhere (Horvat et al., 2007).

### Statistical Analysis

To compare the difference between the two groups, the unpaired Student's *t*-test was used. To compare multiple groups, one-way ANOVA test was performed. GraphPad Prism software was used to determine statistical significance. A *P* < 0.05 was considered significant.

## Results

### NK Cells Are Being Activated and Produce IFN-γ During Secondary Intranasal Infection With *C. muridarum* and Comparable With Primary Infection

**Figure 1:**
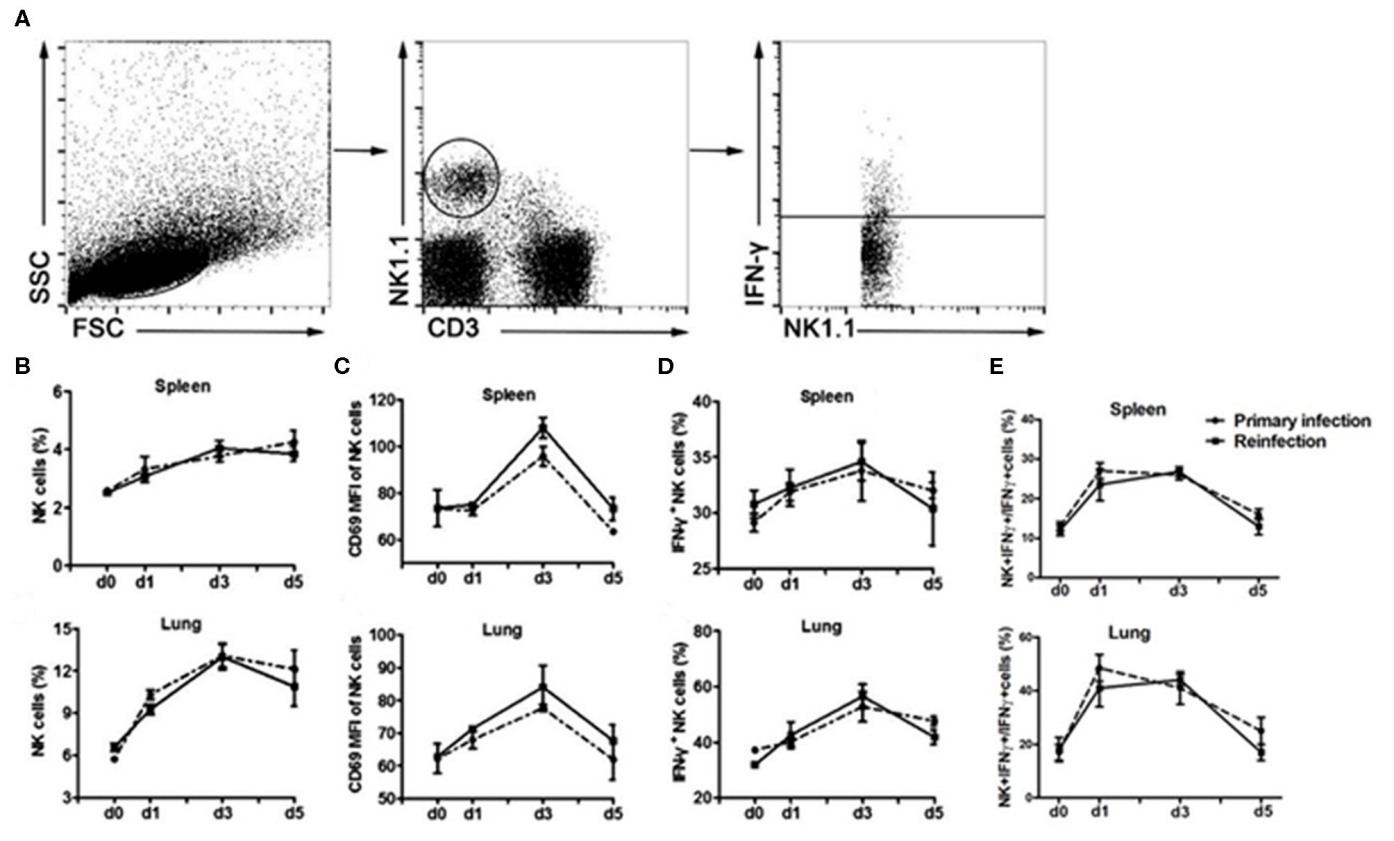
Similar kinetics of NK cells expansion, activation and IFN-γ production following primary or secondary lung infection with *C. muridarum*. Cohort of mice infected intranasally with *C. muridarum* of 1 × 10^3^ IFUs was considered as primary infected mice. In addition, this cohort of mice those were infected intranasally with same dose of *C. muridarum* at 8 weeks after the primary infection is termed here secondary infected mice. Mononuclear cells of spleen and lung were isolated from each group at designated time points, and then labeled with anti- CD3e, -NK1.1, -CD69, and -IFN-γ antibodies for flow cytometric assay. **(A)** Gating strategy used to define NK cells (NK1.1^+^CD3e^−^) and to analyze the IFN-γ production from NK cells. **(B)** Frequency of NK cells in spleen and lung tissue. **(C)** Mean fluorescence intensity (MFI) of CD69 expression on NK cells. **(D)** Frequency of IFN-γ^+^ NK cells. **(E)** Frequency of IFN-γ^+^ NK cells in total IFN-γ^+^ cells. Results are shown as the mean ± SD of three independent experiments (five mice in each group in each experiment) with similar result.

We first evaluated the response of NK cells during the secondary infection of *C. muridarum* (Figure 1). Mice were inoculated intranasally with the same dose of *C. muridarum* at 8 weeks after the primary infection when the bacteria have been cleared from their lung tissues (no bacteria had been detectable at 4 weeks after infection and onwards), and the kinetics of the response of NK cells (NK1.1^+^CD3e^−^) in the spleen and lung tissues at different time points was measured (Figure 1A). The percentages of NK cells started to increase in the spleen as well as lung tissues at day 1 compared to day 0 and continued to rise till day 3 after secondary infection (Figure 1B). CD69 expression on these NK cells also rapidly enhanced following re-infection (Figure 1C). Intracellular cytokine staining analysis showed that the percentage of IFN-γ in NK cells rapidly increased on daya 1 and 3 after infection compared to day 0 (Figure 1D). Notably, the kinetics of expansion of NK cells, the expression of CD69 and IFN-γ in NK cells followed a similar pattern between the primary and the secondarily infected cohort of mice. These results suggest that the secondary infection with *C. muridarum* induces a comparable magnitude of activation and IFN-γ production capacity of NK cells with primary infection.

### Depletion of NK Cells Reduces IFN-γ Production by Memory T Cells Upon *C. muridarum* Antigen Stimulation *in vitro*

**Figure 2:**
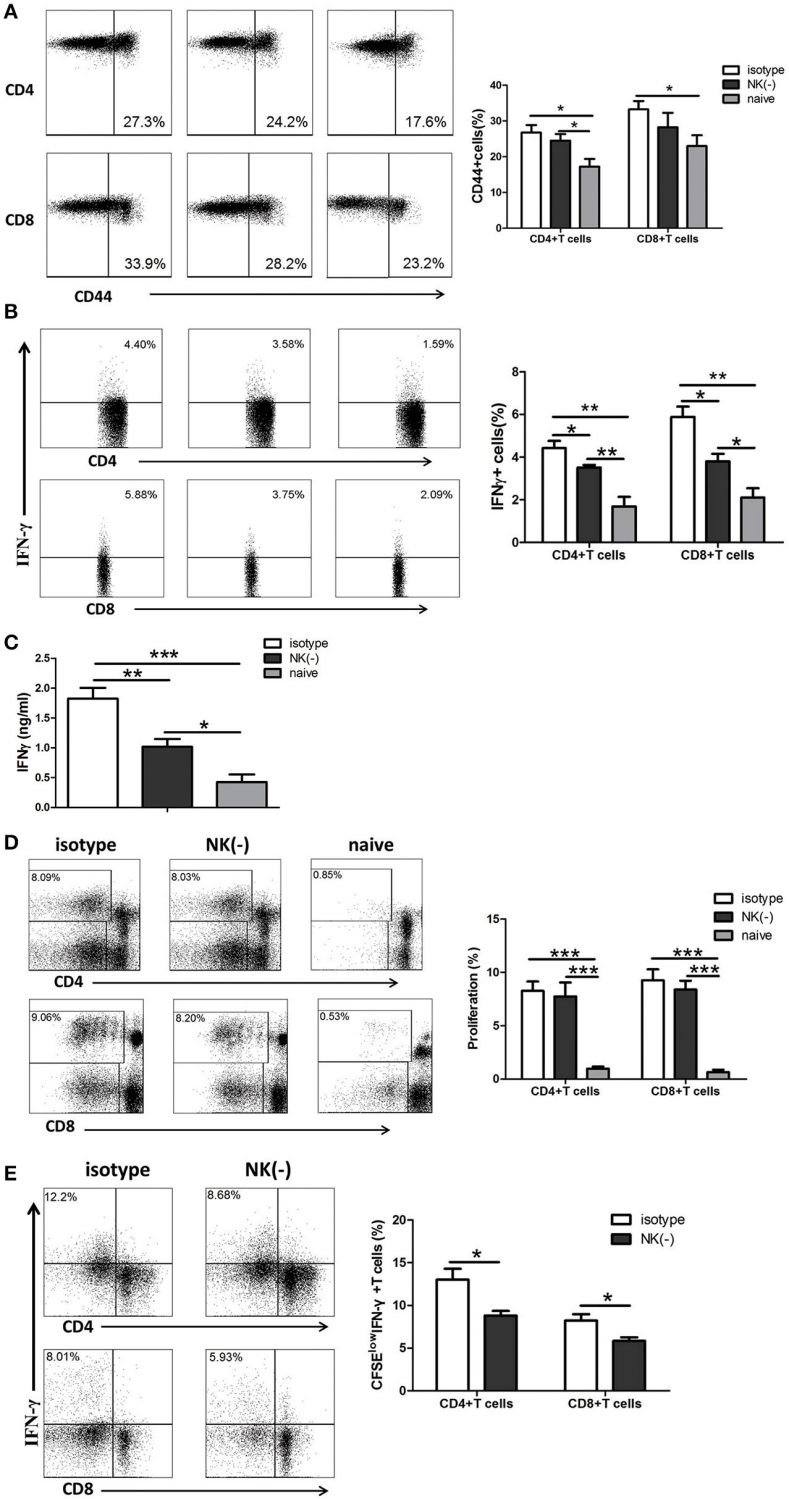
NK cell-depleted mice have reduced production of IFN-γ by memory T cells when stimulated with *C. muridarum* antigen *in vitro*. Eight weeks after primary intranasal *C. muridarum* infection, mice were received twice intravenous injection of normal rabbit IgG (isotype control) or anti–asialo GM1 antibody (20 μl per mice) with the interval of 1 day, and then were killed 2 days after the second injection. Naïve mice without any antibody treatment and infection were used as additional controls. Splenocytes from different groups (five mice in each group) were stimulated with HK*-C. muridarum in vitro* for 72 h **(A–C)**. The expression of CD44 **(A)** and IFN-γ **(B)** within CD4^+^ and CD8^+^ T cells population was determined by flow cytometric assay. IFN-γ secretion in supernatants was assessed by ELISA **(C)**. Splenocytes labeled with CFSE were stimulated *in vitro* with HK*-C. muridarum* for 5 days, and then determined the percentages of proliferating (CFSE ^low^) CD4^+^ and CD8^+^ T cells **(D)** and IFN-γ expression in these cells **(E)**. Data are presented as the mean ± SD of three independent experiments (four to five mice in each group in each experiment) with similar result. ^*^p < 0.05, ^**^p < 0.01, ^***^p < 0.001.

Since IFN-γ is mostly produced by αβ T cells which are the major protective cells in chlamydial lung infection (Yang et al., 1998; Yang, 2003; Joyee et al., 2007, 2008), we investigated the involvement of NK cells in modulating memory T cell responses to chlamydial antigen stimulation *in vitro*. Mice were intravenously administered with anti-asialo-GM1 or isotype control antibodies at 8 weeks after primary *C. muridarum* lung infection. Naïve mice without infection or any treatment were taken as control. Mononuclear cells from spleen were isolated from a different cohort of mice and stimulated with HK-*C. muridarum in vitro* for 72 h. We found that the frequency of activated CD44^+^CD4^+^ or CD44^+^CD8^+^ T cells in mice with NK depleted or non-depleted treatment is comparable, although NK cell-depleted mice showed a trend of a slightly fewer number of activated T cells. However, significantly decreased percentages of IFN-γ^+^ CD4^+^ and CD8^+^T cells were found in NK cell-depleted mice (Figure 2B). Consistently, IFN-γ concentration in the culture supernatants of NK cell-depleted mice was markedly lower than isotype antibody-treated mice (Figure 2C). We further assessed the capacity of the proliferation of these T cells and measured the production of IFN-γ from different groups of mice by labeling their splenocytes with CFSE followed by stimulating with cognate antigen (HK-*C. muridarum)* for 5 days. The proliferation ability of CD4^+^ or CD8^+^ T cells was comparable in mice with and without NK cells depletion (Figure 2D). However, NK cell-depleted mice showed a marked decreased frequency of CFSE^low^ (proliferative) IFN-γ-producing CD4^+^ T cells. A similar pattern of reduced IFN-γ production was observed for CD8^+^ T cells (Figure 2E). Collectively, these *in vitro* experimental data indicate that NK cells have IFN-γ- promoting function on memory T cells.

### NK Cell-Depleted Mice Exhibit Reduced IFN-γ Response After Secondary Infection With *C. muridarum*

**Figure 3:**
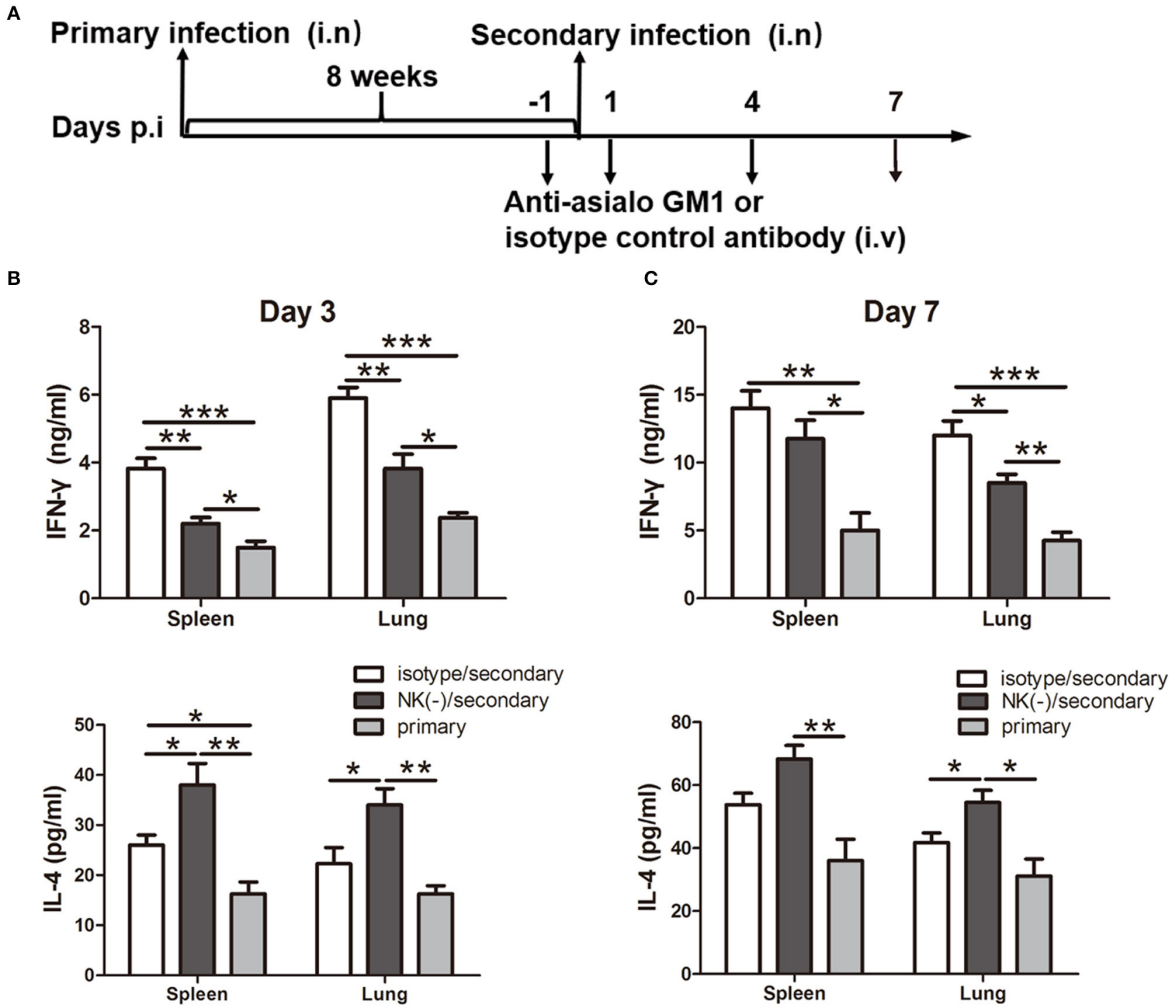
Impact of NK cells depletion on cytokine production induced by chlamydial reinfection. Mice were inoculated intranasally with *C. muridarum* (1 × 10^3^ IFUs) at day 0 for primary infection. Same cohort of mice was infected after 8 weeks of primary infection with same dose of *C. muridarum* was considered secondary infection. Before 1 day and after 1 day of secondary infection, mice were intravenously injected 20 μl anti-asialo GM1 or normal rabbit IgG (isotype control) antibody followed by 10 μl of anti-asialo GM1 or isotype control every 3 days until the mice were killed. Naïve mice received same dose of *C. muridarum* as primary infection control. The schematic diagram of days of infection and antibody treatment **(A)**. At days 3 and 7 after secondary infection, mononuclear cells were isolated from spleens and lung followed by stimulation with HK-*C. muridarum* for 72 h. The concentrations of IFN-γ **(B)** and IL-4 **(C)** in supernatants were tested by ELISA. Results are presented as mean ± SD of three independent experiments (five mice in each group) with similar result. ^*^P < 0.05, ^**^p < 0.01, ^***^p < 0.001.

**Figure 4:**
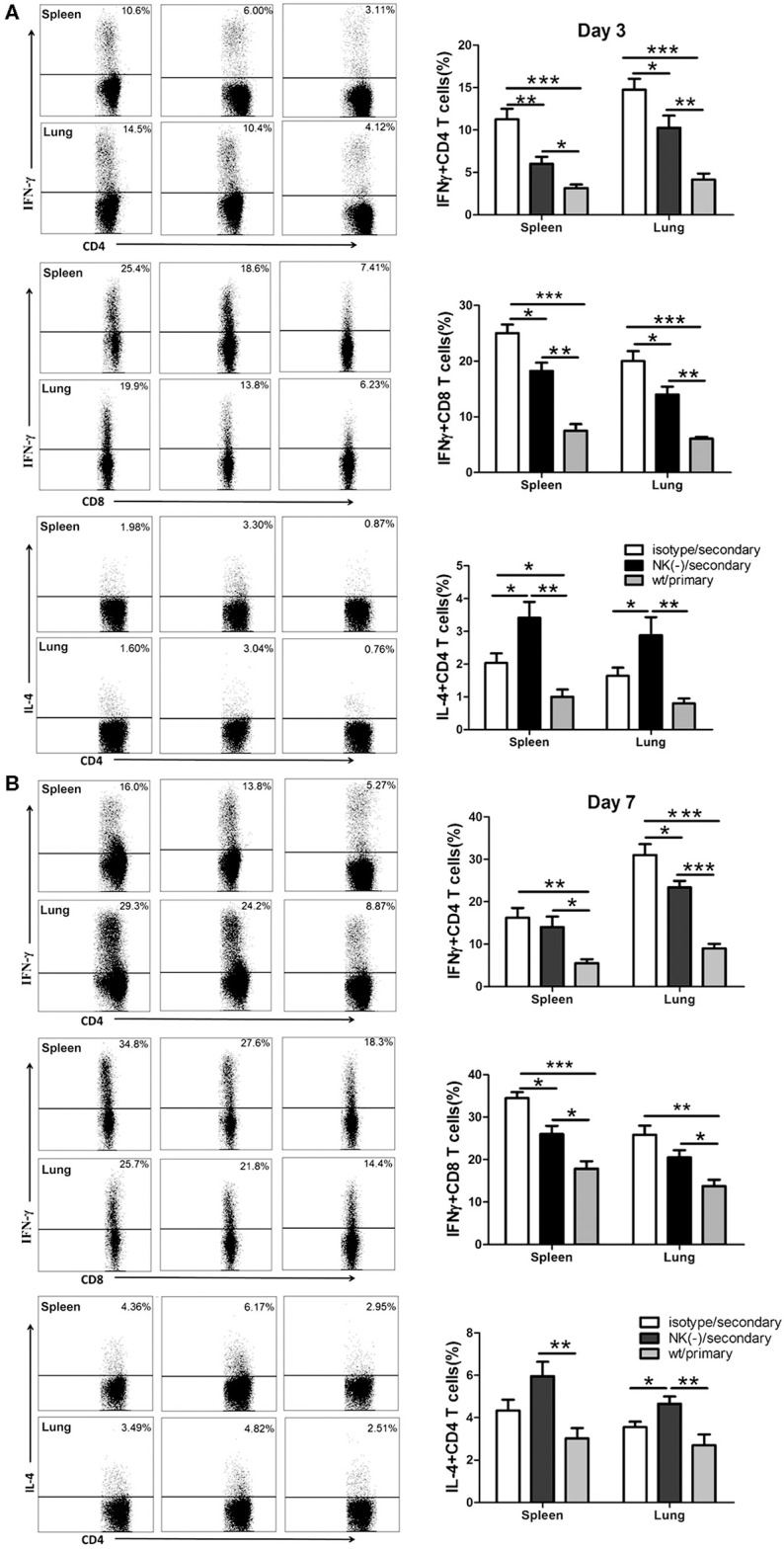
Reduced type 1 T cell responses in NK cell-depleted mice following secondary *C. muridarum* infection. At days 3 and 7 after secondary infection, mice with or without NK cell depletion were sacrificed, and mononuclear cells from spleen and lung were isolated followed by intracellular cytokine staining for flow cytometric detection of cytokines in T cells. The percentage of IFN-γ and IL-4 producing CD4^+^ and CD8^+^ T cells from different cohort of mice at day 3 **(A)** and day 7 **(B)** is shown. Results represent three independent experiments (five mice in each group) with similar result and are shown as mean ± SD. ^*^p < 0.05, ^**^p < 0.01, ^***^p < 0.001.

Having shown the influence of NK cells on memory T cells response using an *in vitro* system, we next examined the involvement of NK cells in recall immune response following secondary infection with *C. muridarum in vivo* (Figure 3A). The cultured supernatants obtained from mononuclear cells from the spleen and lung of NK cell-depleted mice showed significantly reduced IFN-γ secretion especially on day 3 (Figures 3B,C). In contrast, the concentration of IL-4 in these NK cell-depleted mice was higher than NK cell non-depleted mice (Figures 3B,C). The analysis of intracellular IFN-γ production was further performed by flow cytometric assay. Mice with NK depletion displayed significantly lower frequencies of IFN-γ^+^ CD4^+^ or CD8^+^ T cells but a higher percentage of IL-4^+^ CD4^+^ T cells compared to control mice in both spleen and lung tissues at day 3 after secondary infection (Figure 4A). At day 7 following secondary infection, the splenic CD4^+^ T cells showed similar IFN-γ producing capacity between NK cells depleted and non-depleted mice while in lung tissue, CD4^+^ T cells in mice with NK depletion retained the reduced IFN-γ production. NK-depleted mice had reduced IFN-γ-producing CD8^+^ T cells in spleen and lung tissues (Figure 4B). Of note, both NK cells depleted and non-depleted groups with secondary infection displayed markedly higher proportions of T cells expressing IFN-γ than primarily infected mice at days 3 and 7. The results indicate NK cells mediated enhanced type 1 T cell recall responses following chlamydial secondary infection.

### Depletion of NK Cells Enhances Tregs Response in Secondary *C. muridarum* Infection

**Figure 5:**
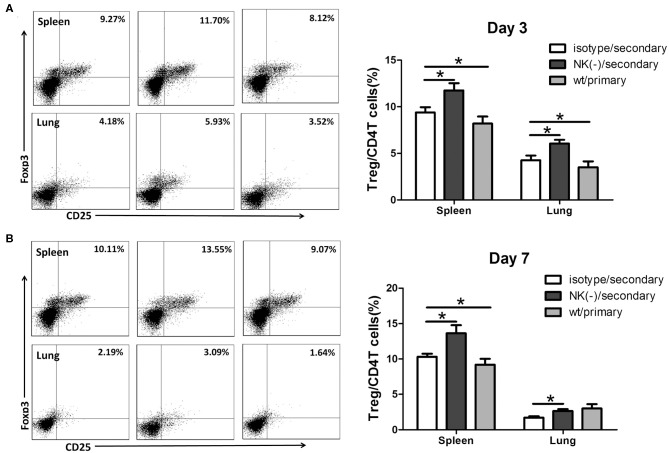
Enhanced Tregs response in NK cell-depleted mice following secondary *C. muridarum* infection. Cohort of mice was killed at days 3 and 7 after secondary infection, and mononuclear cells of spleen and lung were labeled with anti -CD4, -CD25, and -Foxp3 antibodies for flow cytometric assay. Percentages of Treg cells (CD4^+^CD25^+^Foxp3^+^) at day 3 **(A)** and day 7 **(B)** are shown. Data represent three independent experiments (five mice in each group) with similar result and are shown as mean ± SD (*n* = 5). ^*^p < 0.05.

We previously found that NK cells had an inhibitory effect on Tregs response following primary *C. muridarum* infection (Li et al., 2016), we thus examined whether NK cells exhibit the same effect during secondary infection. Mice with NK cell depletion had a significantly higher proportion of CD4^+^CD25^+^Foxp3^+^ T cells (Tregs) than control mice at day 3 (Figure 5A), and this enhanced frequency of Tregs were persisted till day 7 after secondary infection in both spleen and lung tissues (Figure 5B). These findings indicate that NK cells exert an inhibitory effect on Tregs response during secondary *C. muridarum* infection.

### Mice With NK Cell Depletion Display a Reduced Antigen-Specific IgG2a Response Following Secondary *C. muridarum* Infection

**Figure 6:**
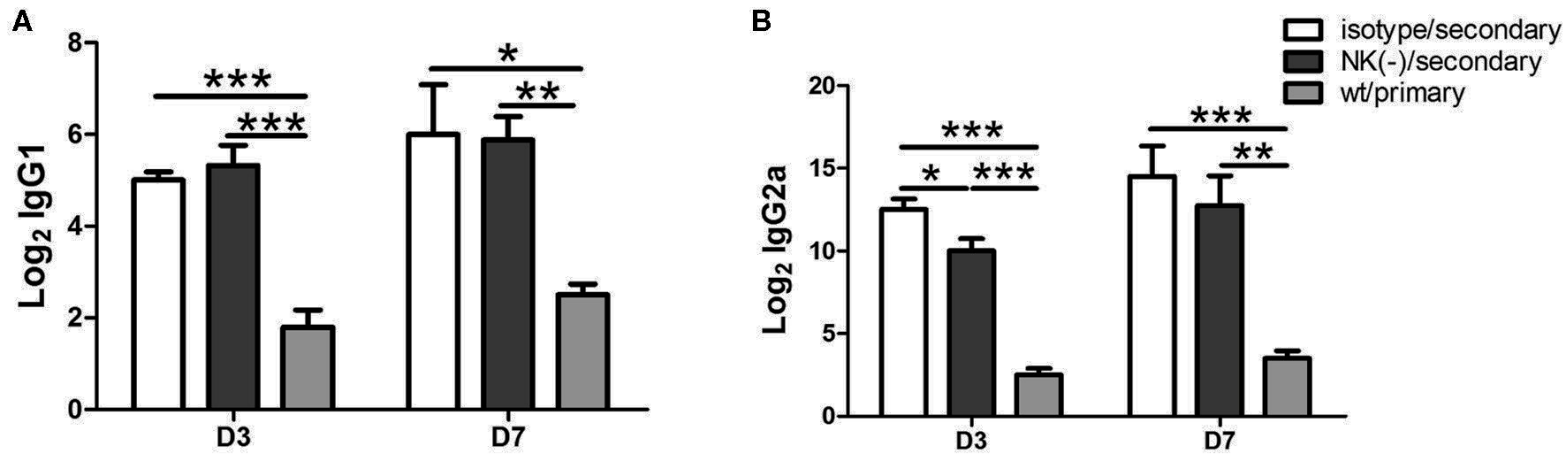
Impact of NK cell depletion on serum antibody responses after *C. muridarum* re-infection. At day 3 and 7 after secondary infection, serum samples were collected from different groups of mice and *C. muridarum*-specific antibody titers of IgG1 **(A)** and IgG2a **(B)** were measured. The results are shown as mean ± SD and represent three independent experiments (five mice in each group) with similar results. ^*^p < 0.05, ^**^p < 0.01, ^***^p < 0.001.

Antibodies play an important protective role during chlamydial secondary infections (Morrison and Morrison, 2005; Li and McSorley, 2015), as well IgG2a, IgG1 antibody is associated Th1, Th2 polarized immune response, we then measured the levels of serum Chlamydia- specific IgG1 and IgG2a antibodies in mice with or without NK cell depletion after secondary infection. The NK cells depleted and non-depleted mice had comparable IgG1 levels (Figure 6A). However, lower levels of IgG2a titer were observed in NK cells depleted mice, particularly at day 3 after secondary infection (Figure 6B). Both groups of mice with secondary infection developed significantly higher titers of Chlamydia-specific IgG2a and IgG1 compared to primarily infected mice. The reduced IgG2a production by NK-depleted mice also suggests an important role of NK cells on type 1 polarization of T cells response with a possible critical role of IFN-γ in IgG2a class-switching.

### Depletion of NK Cells Deteriorates the Protection Against *C. muridarum* in an Early Stage of Secondary Infection

**Figure 7:**
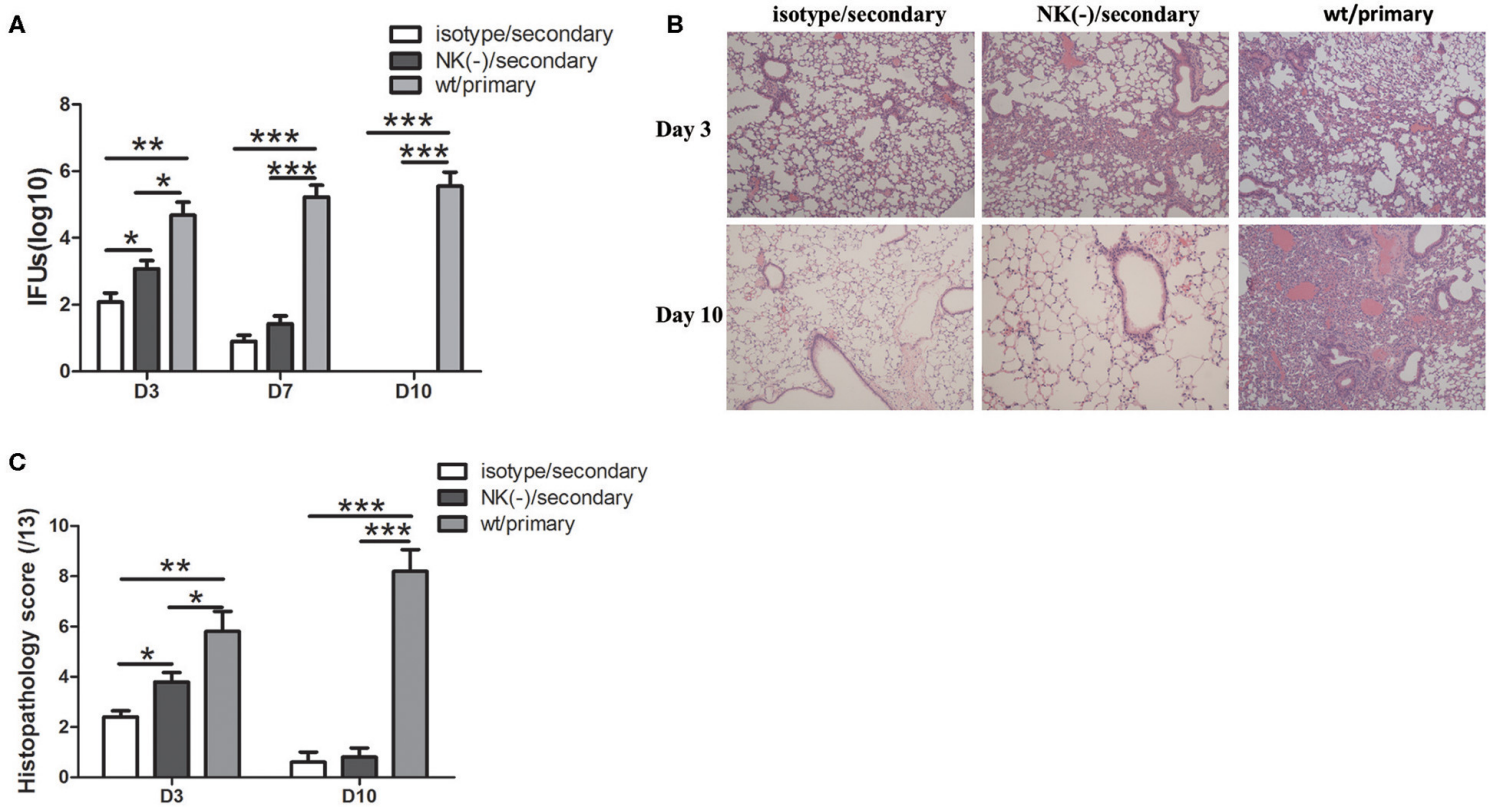
Mice with NK cell depletion exhibited more severe diseases during *C. muridarum* reinfection. Mice reinfected with *C. muridarum* at the same dose (1 × 10^3^ IFU) of primary infection at 8 weeks after primary intranasal infection. Anti–asialo GM1 antibody was used for NK cells depletion as described in Figure 3. At days 3, 7, and 10 after primary and secondary infection, mice were killed and lung tissue homogenate was prepared, used to measure *C. muridarum* loads **(A)**. The lung tissue sections from different groups of mice at days 3 and 10 after primary and secondary infection were stained with H&E and representative images are shown at ×100 magnifications **(B)**. The scores of lung tissue inflammatory grade of histological slides were analyzed as described in Materials and Methods **(C)**. Results are expressed as mean ± SD and represent three independent experiments (five mice in each group in each group) with similar result. ^*^p < 0.05, ^**^p < 0.01, ^***^p < 0.001.

To further establish the role of NK cells in the protective responses, we further examined the effect of these NK cells on the host susceptibility to *C. muridarum* reinfection. Pulmonary bacterial loads in NK-depleted mice were significantly increased compared with those in isotype control at day 3 after secondary infection (Figure 7A). At day 7 after secondary infection, the bacterial loads in both isotype control and NK-depleted mice were rapidly reduced and were comparable. In addition, both of these two groups of mice after secondary infection showed significantly lower chlamydial loads in lung tissues compared to the mice with primary infection on days 3 and 7. At day 10, mice neither with isotype control treatment nor NK cell depletion had any detectable lung *C. muridarum*, while the mice with the primary infection still showed high levels of bacterial loads. We next compared the pathological changes between the two groups of mice in lung tissues after re-infection. Mice with NK cell depletion exhibited more intense pathologic change at day 3. The inflammation in lung tissue almost completely disappeared in both secondary infected groups at day 10; however, the mice with primary infection exhibited severe pathological change and tissue damage (Figure 6B). These data suggest that the NK cells play a protective role during secondary infection with *C. muridarum*.

## Discussion

We have here focused on the contribution of NK cells during secondary lung infection with *C. muridarum*, especially the effect on memory T cell responses. Our results have shown that NK cells play an important role in modulating T cells cytokine production during secondary infection, which correlates with protection. In addition, in recall response, although NK cell depletion has no significant impact on the activation and proliferation of T cells, it markedly decreased the production of IFN-γ by T cells. In secondary infection, mice with NK cells depletion displayed significantly reduced type 1 T cell immunity, however inversely increased Tregs response as well as IL-4 production by CD4^+^ T cells. A lower level of specific IgG2a titer has also been observed following secondary infection in NK cell- depleted mice. More importantly, increased live chlamydial loads and more severe pulmonary pathology have been manifested in mice those have depleted NK cells at early stage of secondary infection with *C. muridarum* (day 3). Results suggest that though NK cells might not be directly required for chlamydial clearance during secondary infection, the presence of these NK cells are crucial to T cells mediated protective recall immunity by cytokine production and inhibition of Tregs response.

It has been well-established that CD4^+^ Th1 response, particularly IFN-γ production, is essential for protective recall immunity to chlamydial infection in animal models (Igietseme et al., 1993; Peterson et al., 1999; Farris and Morrison, 2011; Stary et al., 2015). In accordance with that, recent research on human female subjects who were infected with *C. trachomatis* provided clear evidence that IFN-γ^+^CD4^+^T cells are associated with a protective immune response to *C. trachomatis* reinfection (Bakshi et al., 2018). Supporting with these results, the most important finding of our current study is the induction effect of NK cells on IFN-γ^+^ T cell mediated recall responses which also might answer a part of the question of how these T cells shape their cytokine production in a secondary infection. In the current secondary infection model, NK cells depletion has no significant impact on T cells activation *in vitro* (Figure 2) and *in vivo* (data not shown). In addition, the proliferation of T cells upon re-stimulation with *C. muridarum*-specific antigen was also similar in mice with and without NK cell depletion. However, significantly reduced IFN-γ production by recently proliferating T cells was found in NK cell-depleted mice. The difference of IFN-γ expression by T cells in the two groups was more obvious in the lung than the spleen, consistent with the presence of NK cells in local tissues. The reduced functional capability in terms of IFN-γ production and secretion by antigen-specific T cells in NK cell-depleted mice was further confirmed (Figure 3). These data indicate that NK cells modulate the IFN-γ production by memory T cells. In line with our finding, Habib et al. reported that NK cell depletion led to decreased IFN-γ production by memory T cells, which correlated with reduced protective response upon secondary infection with Ehrlichia (Habib et al., 2016). In addition, it has been reported that NK cells can promote IFN-γ production by memory CTLs against Hepatitis B virus and *Listeria monocytogenes* (Alexandre et al., 2016; Zheng et al., 2016). The influence of NK cells on T cells response was seen mainly at the early stage of secondary infection in this model. NK cell-depleted mice showed a similar pattern of protection like the mice those were not depleted NK cells at day 10 after secondary infection. And also both NK cells depleted and non-depleted mice had lower bacterial load and less pathology compared to primary infection (Figure 7). This suggested that NK cells might play a crucial role in the early stage of T cells recall response. One may argue that lack of apparent influence on long term protection suggests the protective role of NK cell might be less important in secondary infection than primary infection. However, this early NK cell mediated T cells modulation on cytokine production might have long term imprinting on T cells. Therefore, in addition to the molecular mechanism of how NK cells influence the T cells in early stage of infection, the long term imprinting is also a field to explore.

It was also interesting to see in the secondary infection model that an increased Tregs response was induced in mice having depleted of NK cells. The frequencies of Treg cells in spleen and local lung tissues were markedly increased in mice with NK cell depletion following secondary infection. Treg cells are crucial for controlling immune responses against various self and non-self antigens through the direct cell to cell contact or secreting anti-inflammatory cytokines (Rudensky, 2011). The important role of Tregs has been more often observed in primary infections of bacteria, viruses, and fungi (Maloy and Powrie, 2001; Mittrucker and Kaufmann, 2004), as well the effect of Tregs response on recall immunity has also been reported. Tregs depletion in mice immunized with inactivated *C. trachomatis* generated decreased bacterial loads in genital tract tissues after chlamydial challenge (Stary et al., 2015). Moreover, the suppressive effect of Tregs in memory CD8^+^T cells response has been reported previously. For example, Tregs restricted the magnitude of secondary CD8^+^ T cells response to *L. monocytogenes* (Kursar et al., 2002, 2004). Tregs depletion led to increased numbers of protective CD8^+^T memory cells and effective clearance of Herpes simplex virus in DNA-vaccinated mice has been reported (Toka et al., 2004). Furthermore, Treg cells suppressed CD4^+^ memory T cell responses in individuals infected with *Helicobacter pylori* (Lundgren et al., 2003). These data implicate that Tregs might impede in protective recall response to various infections. Our current data has brought a light on the increased response of Tregs in absence of NK cells in *C. muridarum* infection that further demonstrates that this NK cells mediated increased response of Treg cells may be an underlying mechanism of its protective role in secondary *C. muridarum* infection.

The mechanism of how NK cells affecting memory T cells function during secondary chlamydial infection needs more investigation. Our previous studies on primary infection showed that NK cells play its role in manipulating adaptive immunity to *C. muridarum* infection via regulating DC function (Jiao et al., 2011). Recent studies showed that NK-DC interaction also happens during recall responses (Pallmer and Oxenius, 2016; Thomas and Yang, 2016). For example, upon secondary infection with *Listeria*, NK cells were rapidly activated, serving as an important early source of IFN-γ to induce XCR1^+^DCs-mediated reactivation of memory CTLs (Alexandre et al., 2016). Therefore, it is likely that NK cells might impact on T cells recall responses through modulating the function of DCs and other professional antigen-presenting cells. Moreover, NK cells might directly enhance the switch of Th1 cells through producing cytokines including IL-2 and IFN-γ (Habib et al., 2016), although the total number of cells appears not to be influenced by NK cell depletion in this model. Further investigations on the mechanism of how NK cells impacting on protective recall immunity will extend our understanding of the immunobiology of chlamydial infection and will be beneficial for the rational development of the chlamydial vaccine.

Taken together, our findings suggest that NK cells contribute to protective recall immunity against secondary lung infection of *C. muridarum*. The functional contribution of NK cells is likely through promoting type 1 memory T cell responses and restricting Treg cells. The finding provides new insights into the cellular basis of immune responses in secondary chlamydial infection and enhances the understanding of the role of NK cells in immune regulation.

## Data Availability Statement

All datasets generated for this study are included in the article/Supplementary Material.

## Ethics Statement

The animal study was reviewed and approved by Animal Care and Use Committee of Shandong University.

## Author Contributions

XY, WZ, and HW designed the study. JL, XD, and XZ performed the experiments. XW and LZ contributed data analysis in FACS and histopathology. HW drafted the manuscript. XY and RR revised the manuscript. All authors read and approved the final manuscript.

## Conflict of Interest

The authors declare that the research was conducted in the absence of any commercial or financial relationships that could be construed as a potential conflict of interest.
