# Supplementary figures and images for: NK Cells Contribute to Protective Memory T Cell Mediated Immunity to Chlamydia muridarum Infection
Source: Front Cell Infect Microbiol. 2020 Jun 17;10:296. doi: 10.3389/fcimb.2020.00296 (PMC7311576; doi:10.3389/fcimb.2020.00296)

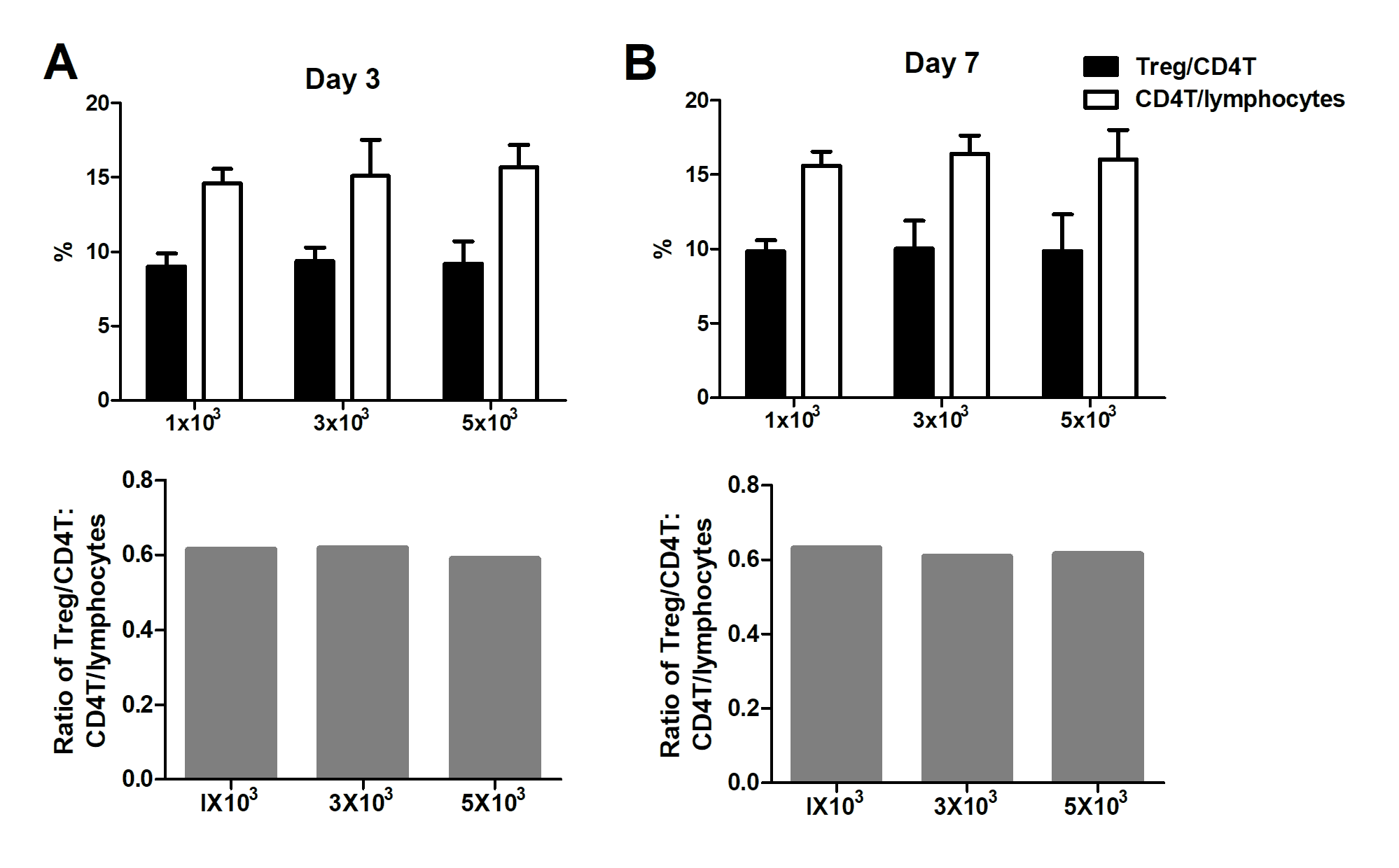

Supplement: Supplementary file 1 [file Image_1.TIF]
