# Supplementary material for: NK Cells Contribute to Protective Memory T Cell Mediated Immunity to Chlamydia muridarum Infection
Source: Front Cell Infect Microbiol. 2020 Jun 17;10:296. doi: 10.3389/fcimb.2020.00296 (PMC7311576; doi:10.3389/fcimb.2020.00296)
Supplement: Supplementary file 2 [file Data_Sheet_1.PDF]

Figure S1. There are no infecting dose dependences in the frequencies of Treg, CD4 T cell or their ratios following secondary *C. muridarum* infection. Mice were inoculated intranasally with 1×10^3 IFUs *C. muridarum*, and then received intranasal reinfection at different dose (1×10^3, 3×10^3 or 5×10^3 IFUs) after 8 weeks of primary infection, respectively. Splenocytes were isolated at day 3 and day 7 during secondary infection followed by flow cytometric detection as described in *materials and methods*. Percentages of CD4 T cells in total lymphocytes, Treg cells in CD4 T cells and the ratios of the mean percentages of these cells at day 3 (A) and day 7 (B) are shown. Results are presented as mean ± SD of three independent experiments (three mice in each group) with similar result.
